# Household Cooking with Solid Fuels Contributes to Ambient PM_2.5_ Air Pollution and the Burden of Disease

**DOI:** 10.1289/ehp.1206340

**Published:** 2014-09-05

**Authors:** Zoë A. Chafe, Michael Brauer, Zbigniew Klimont, Rita Van Dingenen, Sumi Mehta, Shilpa Rao, Keywan Riahi, Frank Dentener, Kirk R. Smith

**Affiliations:** 1 Energy and Resources Group, and; 2 Division of Environmental Health Sciences, University of California, Berkeley, Berkeley, California, USA; 3 School of Population and Public Health, University of British Columbia, Vancouver, British Columbia, Canada; 4 International Institute for Applied Systems Analysis, Laxenburg, Austria; 5 Air and Climate Unit, Institute for Environment and Sustainability, European Commission Joint Research Centre, Ispra, Italy; 6 Global Alliance for Clean Cookstoves, Washington, DC, USA

## Abstract

Background: Approximately 2.8 billion people cook with solid fuels. Research has focused on the health impacts of indoor exposure to fine particulate pollution. Here, for the 2010 Global Burden of Disease project (GBD 2010), we evaluated the impact of household cooking with solid fuels on regional population-weighted ambient PM_2.5_ (particulate matter ≤ 2.5 μm) pollution (APM_2.5_).

Objectives: We estimated the proportion and concentrations of APM_2.5_ attributable to household cooking with solid fuels (PM_2.5-cook_) for the years 1990, 2005, and 2010 in 170 countries, and associated ill health.

Methods: We used an energy supply–driven emissions model (GAINS; Greenhouse Gas and Air Pollution Interactions and Synergies) and source-receptor model (TM5-FASST) to estimate the proportion of APM_2.5_ produced by households and the proportion of household PM_2.5_ emissions from cooking with solid fuels. We estimated health effects using GBD 2010 data on ill health from APM_2.5_ exposure.

Results: In 2010, household cooking with solid fuels accounted for 12% of APM_2.5_ globally, varying from 0% of APM_2.5_ in five higher-income regions to 37% (2.8 μg/m^3^ of 6.9 μg/m^3^ total) in southern sub-Saharan Africa. PM_2.5-cook_ constituted > 10% of APM_2.5_ in seven regions housing 4.4 billion people. South Asia showed the highest regional concentration of APM_2.5_ from household cooking (8.6 μg/m^3^). On the basis of GBD 2010, we estimate that exposure to APM_2.5_ from cooking with solid fuels caused the loss of 370,000 lives and 9.9 million disability-adjusted life years globally in 2010.

Conclusions: PM_2.5_ emissions from household cooking constitute an important portion of APM_2.5_ concentrations in many places, including India and China. Efforts to improve ambient air quality will be hindered if household cooking conditions are not addressed.

Citation: Chafe ZA, Brauer M, Klimont Z, Van Dingenen R, Mehta S, Rao S, Riahi K, Dentener F, Smith KR. 2014. Household cooking with solid fuels contributes to ambient PM_2.5_ air pollution and the burden of disease. Environ Health Perspect 122:1314–1320; http://dx.doi.org/10.1289/ehp.1206340

## Introduction

Approximately 2.8 billion people, more than ever before in human history, use solid fuels, including wood, coal, charcoal, and agricultural residues, for cooking (Bonjour et al. 2013). Solid fuel is usually combusted in inefficient cookstoves, producing a variety of health-damaging gases and particles (Smith et al. 2009), such as black carbon (BC), organic carbon (OC), methane, and carbon monoxide. The 2010 Global Burden of Disease/Comparative Risk Assessment Project (GBD 2010) estimated that exposure to household air pollution from cooking with solid fuels caused 3.5 million premature deaths in 2010 (Lim et al. 2012).

The potential for harm does not stop when this smoke exits house windows or chimneys, however: In areas where solid fuels are the primary source of household cooking, particulate emissions from household cooking with solid fuels contribute significantly to ambient (outdoor) air pollution (Smith 2006). Indeed, the ambient air pollution exposure assessment prepared for GBD 2010 shows substantial exposures occurring in rural areas (Brauer et al. 2012), as do others (Anenberg et al. 2010; Rao et al. 2012). This paper details the methods for calculating the ill health associated with population-wide exposure to just the ambient air pollution caused by household cooking with solid fuels. Together, household and ambient exposure to fine particulate air pollution from household cooking with solid fuels caused an estimated 3.9 million premature deaths in 2010 (Smith et al. 2014), including adjustment for overlaps between the two routes of exposure.

The important contribution of household fuel use (for heating and cooking) to particulate matter emissions has been established in previous emission inventory research. Residential coal and biomass combustion remains a key source of fine particulate matter (≤ 2.5 μm in aerodynamic diameter; PM_2.5_) in China, accounting for 47% (4.3 Tg of 9.3 Tg total) and 34% (4.4 Tg of 13.0 Tg total) of China’s PM_2.5_ emissions in 1990 and 2005 (Lei et al. 2011); the drop in relative contribution was attributable primarily to growth in industrial emissions. Besides industrial processes, energy production and ground transportation are other sectors that contribute substantially to PM_2.5_ pollution.

Recent studies have found that 50–70% of the BC (Cao et al. 2006; Klimont et al. 2009; Lei et al. 2011) and 60–90% of OC emissions in China can be attributed to residential coal and biomass use; Klimont et al. (2009) found similar proportions in India. Even higher contributions were estimated by Ohara (2007): In 2000, 86% of BC emissions in both India and China—together home to more than one-third of the world’s population—could be attributed to residential coal and biomass use; for OC, the proportion was 96% in India and 97% in China.

Source apportionment studies in India and China have shown that biomass combustion can be a major source of ambient particulate air pollution across the urban–rural spectrum (Chowdhury et al. 2012; Wang et al. 2005), despite the observation that household energy use patterns—and associated emissions—tend to differ by population density, economic status, and geographic location (van Ruijven et al. 2011; Zhang et al. 2010). In many countries, solid fuel use is more prevalent in rural areas (Barnes and Floor 1996). However, solid fuels are still used by households in many cities for heating and cooking, as evidenced by the major contributions of biomass burning to urban particulate pollution found in previous source apportionment studies (Health Effects Institute 2010; Pant and Harrison 2012). For the analysis presented here, which focuses on the relative contributions of emission source categories, the exact location of the emission sources is not as significant as it would be for research on individual-level human exposures.

Our objective was to systematically estimate the contribution of household air pollution from cooking with solid fuels (PM_2.5-cook_) to outdoor ambient population-weighted PM_2.5_ air pollution (APM_2.5_), by region, in 1990, 2005, and 2010. Our estimates are based on the fraction of ambient primary combustion-derived household particulate emissions (PPM_2.5-hh_) attributable to cooking and the fraction of APM_2.5_ attributable to household activities (PM_2.5-hh_). These calculations enabled us to estimate the burden of disease from ambient air pollution that can be attributed to household cooking (PM_2.5-cook_), and to better understand the degree to which attainment of outdoor air quality goals depends on control of household air pollution.

We focused specifically on household cooking with solid fuels because this is one of the air pollution risk factors included in GBD 2010. Other household sources of combustion air pollution, including household space heating, were not considered in this analysis. We explored PM_2.5-cook_ at the national level in 170 countries, for the years 1990, 2005, and 2010, and report the results at the regional level in concordance with GBD 2010 [Brauer et al. 2012; Institute for Health Metrics and Evaluation (IHME) 2010].

The main data sources used in this analysis were *a*) emissions estimates from the Greenhouse Gas and Air Pollution Interactions and Synergies (GAINS) models hosted by the International Institute of Applied Systems Analysis (IIASA) in Laxenburg, Austria (http://gains.iiasa.ac.at/models/index.html) (Amann et al. 2011; Cofala et al. 2012) and *b*) atmospheric concentration estimates from the TM5-FASST (Fast Scenario Screening Tool for Global Air Quality and Instantaneous Radiative Forcing, paired with TM-5, a global chemical transport model) screening tool hosted by the European Commission Joint Research Center (JRC) based on emissions estimates from the Model for Energy Supply Strategy Alternatives and their General Environmental Impact (MESSAGE) (Rao et al. 2012).

## Methods

Because most emission inventories report total residential emissions (Bond et al. 2004; Lamarque et al. 2010; Shen et al. 2012; Streets et al. 2003) with no distinction between cooking and heating, our general approach was to calculate *a*) the proportion of PM_2.5-hh_ emissions attributable to cooking (rather than heating), and then *b*) the proportion of APM_2.5_ attributable to PM_2.5-hh_. To focus specifically on the residential sector, we used GAINS and Equation 1 to determine the fraction of PPM_2.5-hh_ from cooking with solid fuels such as hard coal, agricultural residues, fuelwood, and dung, for each country or subnational jurisdiction (IIASA 2012):

(PIT + STOVE)/ΣDOM = PPM_2.5-hh_ from cooking, [1]

where PIT indicates emissions from open fire cooking with solid fuels (teragrams of PPM_2.5_ per country), STOVE represents emissions from combusting solid fuels in residential cooking stoves (teragrams of PPM_2.5_ per country), and DOM indicates total emissions from all residential sources, including boilers and heating stoves (teragrams of PPM_2.5_ per country). Non-fuel emissions associated with cooking (such as volatile organic compounds created by frying) are not included.

Within GAINS, we used a scenario that draws on data from the International Energy Agency (IEA 2011). GAINS estimates current and future PPM_2.5_ emissions using activity data, fuel-specific uncontrolled emission factors, the removal efficiency of emission control measures, and the extent to which such measures are applied (Amann et al. 2011; Kupiainen and Klimont 2007). For household cooking with solid fuels from 1990 through 2010, no technical control measures were applied in the model.

We multiplied the fraction of residential PPM_2.5_ attributable to household cooking by the proportion of total ambient population-weighted PM_2.5_ attributable to household combustion (PM_2.5-hh_) (Equation 2). The latter proportion (%PM_2.5-hh_) was generated using TM5-FASST.

%PPM_2.5-hh_ from cooking × %PM_2.5-hh_ = %PM_2.5-cook_, [2]

where all analysis in this equation is at the country level, %PPM_2.5-hh_ from cooking is the quantity derived in Equation 1, and %PM_2.5-hh_ = μg/m^3^ PM_2.5-hh_/μg/m^3^ PM_2.5_.

Equation 3 shows the method by which country-level results were combined to produce regional population-weighted estimates.


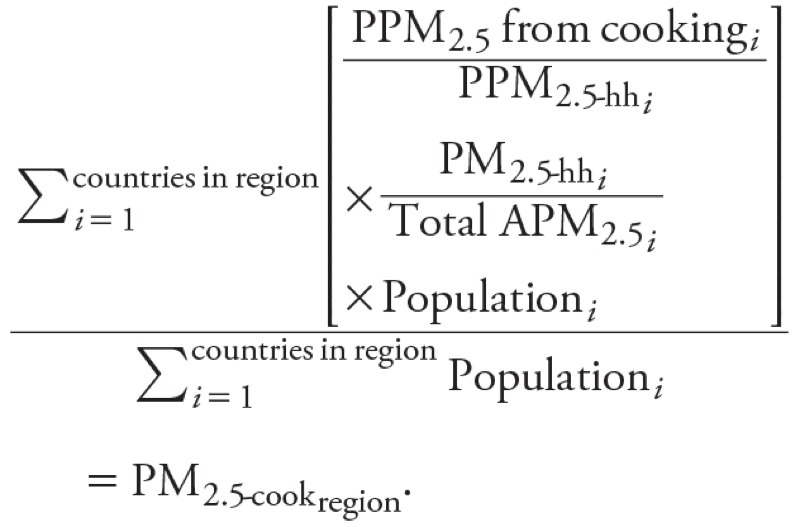
[3]

We used global estimates of annual average ambient population-weighted PM_2.5_ concentrations, which were developed for the GBD 2010 study (Brauer et al. 2012) as well as the Global Energy Assessment (Riahi et al. 2012), to estimate the proportions and absolute concentrations of PM_2.5-cook_, on a regional basis. The underlying methodology for deriving PM_2.5_ concentrations is described in Rao et al. (2012) and combines the global integrated assessment model MESSAGE (Rao and Riahi 2006; Strubegger et al. 2004) with TM5 (see Supplemental Material, “Model Methodologies”). MESSAGE covers all greenhouse gas–emitting sectors; in the residential sector, MESSAGE includes an explicit representation of the energy use of rural and urban households with different income levels. Fuel choices at the household level consider the full portfolio of commercial fuels as well as traditional biomass for cooking, heating, and specific use of electricity of household appliances (Ekholm et al. 2011). TM5-FASST was used to determine PM_2.5-hh_. Secondary organic aerosol formation was included in TM5-FASST estimates of annual average population-weighted PM_2.5_ concentrations (see Supplemental Material, Figure S1, for more information on the emission and source categories included in this analysis). Dust and sea salt increments were estimated by comparing concentrations generated by TM5-FASST with those developed with TM5-FASST, satellite data, and ground measurements for GBD 2010 and published by Brauer et al. (2012). Positive differences between GBD 2010 and TM5-FASST were assumed to be representative of dust and sea salt increments and were included in estimates of APM_2.5_ to better approximate the proportional role of household solid fuel use for cooking in creating APM_2.5_.

Following GBD 2010 (IHME 2010), this analysis considers PM_2.5_ emissions for three time points: 1990, 2005, and 2010. The data cover 170 countries (see Supplemental Material, Table S1) in 20 of the 21 GBD 2010 regions; the majority of missing countries are small (population < 1 million each) and together they account for 34 million people in 2010, that is, < 1% of the world population.

Data sources and models used in our analysis are summarized in Table 1. Regional population and household emissions estimates are shown in Supplemental Material, Table S4.

**Table 1:** Sources of input data.

Data source and model	Purpose in this analysis	Data attributes	Spatial resolution	References
GAINS	Calculate proportion of household PM_2.5_ emissions that comes from cooking	Includes household cooking stoves and open-pit cooking emissions. Does not include nonfuel cooking emissions. Units: mass emissions of primary PM_2.5_, by sector and technology used.	Country or subcountry	IIASA 2012; IEA 2011; Purohit et al. 2010
TM5-FASST (MESSAGE)	Calculate proportion of ambient PM_2.5_ that comes from household combustion	Uses MESSAGE to calculate particulate matter emissions by sector and TM5 atmospheric chemical transport model to calculate secondary organic aerosol formation. Units: concentrations (μg/m^3^) of annual average population-weighted PM_2.5_. Includes secondary organic aerosol formation. Dust and sea salt estimated by comparing combustion-derived PM_2.5_ to total ambient PM_2.5_ reported by Brauer et al. (2012).	Country or region (derived from gridded 1° × 1° concentration results)	Brauer et al. 2012
Global burden of disease	Calculate ill health resulting from exposure to outdoor PM_2.5_ air pollution	Uses estimates of average annual population-weighted PM_2.5_ concentrations to calculate ill health from outdoor air pollution. Units: annual deaths and DALYs, by region.	Deaths and DALYs: region PM_2.5. _Concentrations: 0.1° × 0.1° gridded	Brauer et al. 2012; Lim et al. 2012

We estimated the burden of disease associated with exposure to outdoor PM_2.5_ air pollution that can be attributed to household cooking by applying the derived proportions of APM_2.5_ due to household cooking with solid fuels to the GBD 2010 burden of disease estimates for ambient air pollution (Lim et al. 2012). We scaled results—that is, we applied percentages of ambient air pollution due to household cooking with solid fuels (the risk factor) to the burden estimates while preserving the exposure–response relationships used to determine the overall burden of disease attributable to ambient air pollution.

## Results

**Figure 1:**
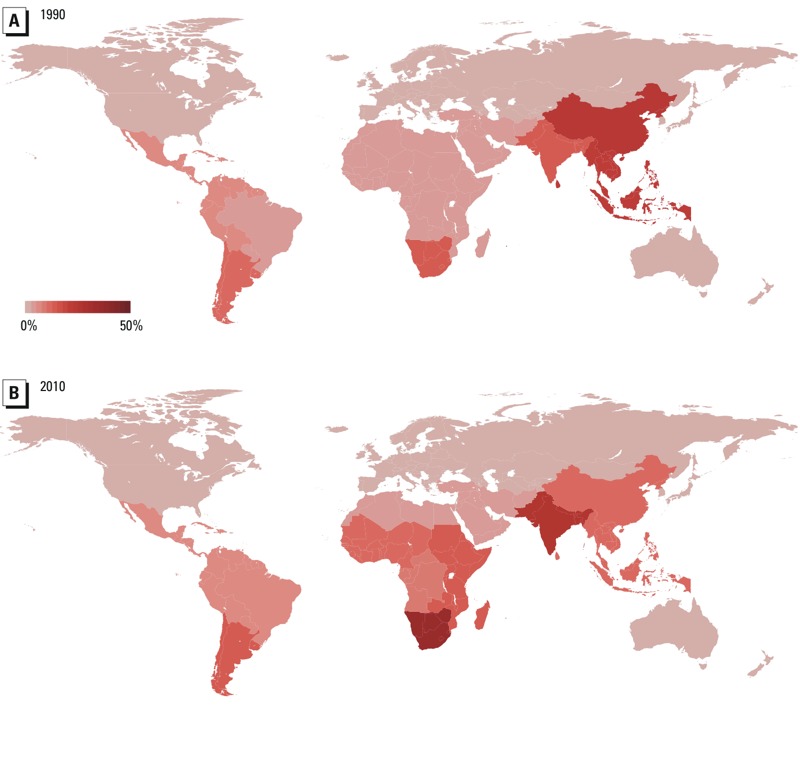
Percentage of population-weighted ambient PM_2.5_ attributable to household cooking with solid fuels, 1990 (*A*) and 2010 (*B*).

Globally, we estimated that about 12% of population-exposure weighted average ambient PM_2.5_ is attributable to household use of solid cooking fuels (Table 2, Figure 1). In 7 of the 20 regions analyzed, at least 10% of ambient PM_2.5_ was attributed to household cooking in 2010. These 7 regions encompass 41 countries and are home to > 4 billion people. In contrast, 7 of the regions analyzed (representing 56 countries with 1.4 billion people) had negligible levels (< 2% PM_2.5-cook_) throughout the 1990–2010 study period. By region, estimated proportions of APM_2.5_ attributable to PM_2.5-cook_ in 2010 ranged from 0 to 37% (Figure 1). In general, we observed that an increase in country-level economic status was accompanied by a decrease in the contribution of household cooking to APM_2.5_.

**Table 2:** Population-weighted contribution of cooking to ambient particulate matter pollution (PM_2.5-cook_), by region.

GBD 2010 region^*a*^	PM_2.5-cook_ (%)^*b*^			PM_2.5-cook_ (μg/m^3^)^*c*^			APM_2.5_^*d*^		
	1990	2005	2010	1990	2005	2010	1990	2005	2010
Southern sub-Saharan Africa	13.0	32.0	37.0	0.8	2.2	2.8	6.4	6.6	6.9
South Asia	15.0	30.0	26.0	4.4	9.4	8.6	30.0	32.0	33.0
Southern Latin America	11.0	13.0	15.0	0.8	0.8	1.0	6.4	6.0	5.9
Eastern sub-Saharan Africa	4.9	12.0	13.0	0.5	1.1	1.2	11.0	12.0	12.0
Southeast Asia	22.0	13.0	11.0	3.9	2.5	2.0	16.0	17.0	17.0
East Asia	23.0	14.0	10.0	11.0	9.1	7.3	49.0	63.0	72.0
Western sub-Saharan Africa	3.4	9.0	10.0	0.9	2.2	2.4	27.0	27.0	27.0
Central sub-Saharan Africa	3.7	9.4	9.8	0.6	1.3	1.4	16.0	14.0	14.0
Tropical Latin America	3.9	6.2	7.1	0.2	0.3	0.4	5.2	5.2	5.1
Andean Latin America	5.7	5.2	5.7	0.4	0.4	0.4	7.8	8.2	8.0
Central Latin America	5.5	5.0	5.3	0.7	0.5	0.5	14.0	11.0	12.0
Caribbean	7.1	4.7	5.3	0.6	0.4	0.4	8.6	9.3	9.1
North Africa and Middle East	3.3	3.8	3.3	0.9	1.0	0.9	30.0	29.0	29.0
High-income Asia Pacific	1.1	0.6	0.6	0.3	0.2	0.2	31.0	27.0	26.0
Central Asia	0.9	0.1	0.2	0.1	0.0	0.0	24.0	21.0	20.0
Australasia	0.0	0.1	0.0	0.0	0.0	0.0	5.0	5.0	5.7
Western Europe	0.0	0.0	0.0	0.0	0.0	0.0	25.0	17.0	15.0
Central Europe	0.0	0.0	0.0	0.0	0.0	0.0	31.0	19.0	16.0
Eastern Europe	0.0	0.0	0.0	0.0	0.0	0.0	19.0	10.0	10.0
High-income North America	0.0	0.0	0.0	0.0	0.0	0.0	18.0	13.0	13.0
Global	11.0	13.0	12.0	4.0	4.5	4.0	29.0	30.0	31.0
^***a***^Regional groupings, defined by IHME for the Global Burden of Disease 2010 project, are described in Supplemental Material, Table S1. ^***b***^Percent of population-weighted annual average ambient PM_2.5_ attributable to household cooking. ^***c***^Concentration of population-weighted annual average ambient PM_2.5_ attributable to household cooking (μg/m^3^). ­^***d***^Concentration of total population-weighted annual average ambient PM_2.5_ (μg/m^3^).									

**Figure 2:**
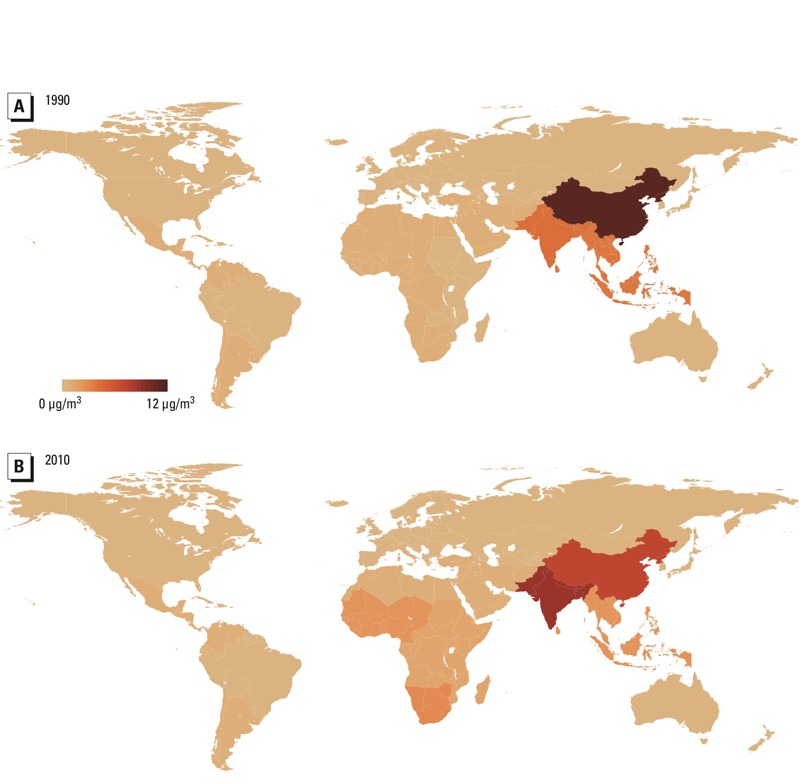
Population-exposure weighted concentration of ambient PM_2.5_ attributable to household cooking with solid fuels, 1990 (*A*) and 2010 (*B*).

Between 1990 and 2010, East Asia (including China) experienced a decline in absolute levels of PM_2.5-cook_ (from 11 to 7 μg/m^3^) (Figure 2) as well as a decline in the percent of PM_2.5_ from cooking (from 23% to 10% in 2010) (Figure 1). This occurred alongside a global increase in ambient PM_2.5_ concentrations: Brauer et al. (2012) reported that population-weighted regional annual average PM_2.5_ concentrations rose between 1990 and 2010 in most parts of Asia, including East Asia (from 49 μg/m^3^ in 1990 to 72 μg/m^3^ in 2010), while falling in North America and Europe, including Central Europe (31 μg/m^3^ in 1990, 16 μg/m^3^ in 2010).

Overall, the estimated population-weighted global annual average PM_2.5_ concentration rose slightly from 29 to 31 μg/m^3^ over this period. This was driven partly by increases in household cooking emissions in South Asia, which includes India: Although the percentage of PPM_2.5-hh_ attributable to cooking remained steady around 82% between 1990 and 2010 (see Supplemental Material, Table S4), PM_2.5-cook_ rose from 15% to 26%, or 4 μg/m^3^ to 9 μg/m^3^ (Table 2), while APM_2.5_ rose from 30 μg/m^3^ to 33 μg/m^3^.

The APM_2.5_ formed by household cooking emissions has major implications for human health, as well as outdoor and indoor air quality. Worldwide, the use of solid fuels for household cooking is estimated to have resulted in 370,000 deaths and 9.9 million disability-adjusted life years (DALYs) in 2010 (Table 3). The vast majority of these deaths were in South Asia (200,000), which includes India, and East Asia (130,000), which includes China. The relative decrease in PM_2.5-cook_ in East Asia from 1990 through 2010 (Table 2), which was estimated to result in 90,000 fewer deaths per year (Table 3), was more than offset by an estimated increase of 121,000 deaths per year from exposure to PM_2.5-cook_ in South Asia over the same time period.

**Table 3:** Estimated burden of disease from exposure to ambient PM_2.5_ attributable to household cooking with solid fuels.

GBD 2010 region^*a*^	Deaths			DALYs		
	1990	2005	2010	1990	2005	2010
South Asia	79,000	210,000	200,000	3,100,000	6,700,000	6,000,000
East Asia	220,000	170,000	130,000	5,700,000	3,700,000	2,600,000
Southeast Asia	24,000	20,000	18,000	800,000	510,000	450,000
Western sub-Saharan Africa	2,400	6,300	7,800	140,000	320,000	380,000
North Africa and Middle East	4,500	6,200	5,800	150,000	170,000	160,000
Eastern sub-Saharan Africa	1,400	3,200	3,500	74,000	150,000	140,000
Central sub-Saharan Africa	480	1,300	1,600	24,000	53,000	65,000
Central Latin America	1,200	1,100	1,400	37,000	26,000	33,000
Southern sub-Saharan Africa	330	1,000	1,400	11,000	36,000	41,000
Tropical Latin America	240	480	540	6,800	12,000	13,000
High-income Asia Pacific	840	470	530	17,000	7,800	8,200
Southern Latin America	440	440	500	9,800	9,000	9,900
Caribbean	390	330	380	9,900	7,500	8,700
Andean Latin America	140	140	160	5,500	3,900	4,200
Central Asia	490	51	78	16,000	1,400	2,000
Western Europe	150	4	2	2,400	64	24
Australasia	0	1	1	4	9	9
Central Europe	0	0	0	0	0	0
Eastern Europe	0	0	0	0	0	0
High-income North America	0	0	0	0	0	0
Global	330,000	420,000	370,000	10,000,000	12,000,000	9,900,000
^***a***^Regional groupings, defined by IHME for the GBD 2010 project, are described in Supplemental Material, Table S1.						

Despite the high proportion of APM_2.5_ attributable to household cooking in Southern sub-Saharan Africa, the estimated health impacts from resulting ambient air pollution exposures were relatively modest (41,000 DALYs in 2010) (Table 3). However, across the four sub-Saharan African regions, estimated annual deaths due to exposure to APM_2.5_ from cooking more than doubled (Eastern sub-Saharan Africa), tripled (Central and Western sub-Saharan Africa), or quadrupled (Southern sub-Saharan Africa) between 1990 and 2010.

## Discussion

Although all household cooking contributes to ambient air pollution, either directly at the household level, through production and transport of fuel, or indirectly through the manufacture of cooking technologies, we estimated only particulate emissions from the combustion of solid fuels in the household. Kerosene, for example, creates BC and other particulate matter at the point of use (Lam et al. 2012b), and even electric cooking contributes indirectly to air pollution through emissions at power plants, but these emissions were not counted in the present analysis.

In addition, we made the following important assumptions in our analysis:

- Isolating household cooking emissions. We assumed that household cooking emissions are correctly split from commercial cooking emissions, although we realize that there is often an overlap between these two categories. We also assumed that energy use and emissions databases (GAINS and MESSAGE) and their underlying data sources correctly characterize the split between fuels used for household cooking and those used for household heating, although we realize that cooking and heating energy use may overlap. IIASA collaborates with partners in China, India, and Pakistan and uses published sources of information (local reports and peer reviewed research), as well as regional GAINS studies (Amann et al. 2008; Purohit et al. 2010) to distinguish household fuel use for heating from that for cooking, especially in northern China. In a number of countries in Asia, GAINS allocates activities also at the subnational level, for example, provinces in China or India. The split between cooking and heating in Europe was developed using data from European Commission consultations under the Convention for Long Range Transboundary Air Pollution.
- Escape fraction. We assumed that the particle escape fraction is 100%; that is, all particles generated by combustion inside a home or cooking structure are eventually incorporated into ambient air, and there is no significant mass loss due to particle deposition on indoor surfaces. Although little work has been done to characterize the fate of indoor combustion particles and their flow out of enclosed spaces, modeling estimates show that approximately 90% of fine particles are likely to reach the outdoor environment, a figure that probably rises to nearly 100% in houses with high air exchange rates (Lam et al. 2012a). In addition, many households cook outdoors for at least part of the year.
- Atmospheric transformation. GAINS data are presented in units of mass of PPM_2.5._ We assumed that all primary particulate household emissions contribute in the same way to total PM_2.5;_ that is, each gram of PPM_2.5-cook_ will eventually create the same mass of PM_2.5_ (after atmospheric interactions) as will any other gram of PPM_2.5-hh_.
- Atmospheric transport. We assumed that PM_2.5_ concentrations attributed to household emissions result solely from particles emitted from households inside the country/region in question, without notable contribution (via atmospheric transport) from neighboring regions.
- Spatial misalignment. We assumed that the proportion of ambient PM_2.5_ attributable to PM_2.5-cook_ is uniform across a given country. Although we recognize that there can be much local variation in the degree to which household fuels contribute to ambient PM_2.5_, we made this assumption based on the spatial scale at which emissions are reported, which, in the case of this globally consistent analysis, is at the country or regional level. The analysis reported here was performed at the country level (and is reported at the regional level). We were not able to systematically account for urban–rural differences in population density, household solid fuel use, or exposure to ambient air pollution within countries because of data limitations. We attempted to generate sensitivity analysis estimates at the urban–rural level, but inconsistencies among available international databases at this spatial scale introduced substantial unexplained variation. Currently, the definition of urban–rural areas is not consistent across countries or data sources. We concluded that the consequent loss of comparability, and difficulty of explaining the variations, obviated any improvement in estimated values that might have occurred in some countries.

*Emissions estimates*. This analysis used multiple emissions information sources with different system boundaries (see Supplemental Material, Tables S2–S3 and Figure S1). The GAINS model provides estimates of PPM_2.5-hh_; TM5-FASST provides estimates of APM_2.5_ by source category, including primary combustion-derived emissions and secondary particulate formation. Neither model includes salt or dust emissions, though dust and sea salt were estimated by comparing combustion-derived PM_2.5_ from TM5-FASST with APM_2.5_ estimates developed for GBD 2010 (Brauer et al. 2012) and used in the burden estimates (Lim et al. 2012).

Insufficient input data made it challenging to conduct this analysis for some parts of the world, notably the eight sub-Saharan African and Latin American GBD regions. Regional assumptions about emissions patterns were made when country-level data were not available, and emission factors were often estimated within one country and applied to other countries when country-specific emissions data were not available.

Many countries, including India and China, lack the detailed national emission inventories that are available in the United States, Canada, and most European countries (Lei et al. 2011). Household cooking data remain scarce and relatively poor in quality, owing to the difficulties of measuring household fuel use in developing countries and emerging economies. From household survey questions that are too general to generate accurate projections, to emission factors that are sensitive to local meteorological or fuel conditions (such as wood moisture content), to poor data on emerging control strategies (such as advanced biomass cookstoves), the data used to create the results presented here have weaknesses. Furthermore, as noted above, the lack of urban and rural disaggregation of energy use and sectoral emissions data make it difficult to account for demographic trends that may influence exposure.

In addition to improving household energy use and emission estimates, there is a need to work toward more comprehensive data harmonization and sharing in this specific issue area. Major emissions inventories and models continue to use different household fuel use inputs (Fernandes et al. 2007; Klimont et al. 2009; Pachauri 2011), so results are not directly comparable across models, although efforts to improve this issue are underway (Bonjour et al. 2013). This methodology represents a first attempt to generate globally commensurate estimates of the contribution of household cooking to ambient air pollution, but there is a need to improve upon this analysis as better data sources become available.

*Uncertainty of emissions estimates and atmospheric chemistry models*. Even when well-supported energy use information exists, there is a great deal of uncertainty associated with particulate emissions estimates, partly because emission factors vary with specific fuel type, fuel quality, and combustion conditions [United Nations Energy Program (UNEP) 2011]. Household fuel use emissions estimates, especially from coal combustion, are more uncertain than estimates of emissions from other sectors, because of the range of combustion conditions and fuels used; one of the many reasons for this uncertainty is that laboratory experiments designed to understand household stove emissions often produce different results than those measured in the field (Jetter et al. 2012). Uncertainties around estimates of BC and OC emissions are notoriously high: in an analysis of the INTEX-B (Intercontinental Chemical Transport Experiment–Phase B) Asian emissions inventory, which used a similar modeling technique to the GAINS model used here, uncertainty around BC and OC emissions (± 208–364%, ± 258–450%) was found to be an order of magnitude greater than for some other air pollutants [sulfur dioxide, nitrogen oxides (NO_x_)] (UNEP 2011; Zhang et al. 2009). The uncertainty around undifferentiated PM_2.5_ was somewhat smaller (± 130%) (Zhang et al. 2009).

Atmospheric chemistry transport models have their own uncertainties, related to chemistry, dispersion, and removal of aerosol. For instance, intercomparisons of global models have shown that even when the same emission inventories were used, a large range of aerosol global properties were seen (Huneeus et al. 2011; Textor et al. 2006). However, the specific combination used in this analysis—of GAINS emissions and chemical transport model TM5—was tested and compared with a global data set of PM_2.5_ observations, as well as an independent study that combined MISR/MODIS (Multi-angle Imaging SpectroRadiometer/Moderate Resolution Imaging Spectroradiometer) satellite columns with assumed vertical aerosol distributions from the global GEOS-Chem (Goddard Earth Observing System) model (Brauer et al. 2012). Both studies showed a rather favorable comparison to outdoor PM_2.5_ measurements, with relative errors in the order of ± 10% in the range of 10–200 μg/m^3^.

Because we examined household emissions rather than human exposures, we probably underestimated the magnitude of associated health effects, for two reasons: First, household emissions vary seasonally (as do overall PM_2.5_ emission levels and the specific composition of PM_2.5_), and often peak in the winter in much of Asia and probably many other regions (Chowdhury et al. 2007; Stone et al. 2010). During the heating season, a particularly pronounced increase in mortality risk associated with exposure to secondary aerosols and combustion species has been documented in China (Huang et al. 2012). Second, household emissions probably have a higher average intake fraction than most sources of ambient air pollution, because people spend long hours in very close proximity to cooking and heating stoves; the intake fraction may, in urban areas, be on par with that of electric generators, construction equipment, and vehicles (Apte et al. 2011; Bennett et al. 2002; Health Effects Institute 2010), though vehicles produce less primary PM_2.5_ than households, in many countries, as noted below. In general, there is a pressing need for more research on sector-specific contributions to exposure and disease burden, rather than emissions or concentrations of air pollutants.

*Technology and policy implications*. Solid fuels are expected to remain an important source of energy for household cooking for decades to come (Global Energy Assessment 2012; Pachauri 2011). Although the demand for wood as a cooking fuel generally decreases with economic growth (Smith et al. 1994), and emissions can be partially controlled with the use of certain advanced cookstoves (Jetter et al. 2012), this decline may be offset by a trend toward smaller families, which tends to raise per capita solid fuel consumption (Knight and Rosa 2011).

More than half of the world’s population lives in areas where household cooking significantly affects air quality. Our results indicate that it will be difficult to reduce ambient PM_2.5_ to meet air quality standards unless household emissions are addressed, along with other sources (Balakrishnan et al. 2011). On-road cars, trucks, and other transport vehicles are more widely recognized as sources of ambient air pollution, compared with household cooking emissions, especially in industrialized countries (Bond et al. 2004, 2013; Kupiainen and Klimont 2007; UNEP 2011). However, direct PM_2.5_ emissions associated with on-road transport are often much lower than the less well-known and more dispersed problem of PM_2.5-cook_, something that has been noted in other analyses as well (Lei et al. 2011); however, vehicles do contribute higher levels of other air pollutants, such as NO_x_. Similarly, although not addressed here, in many temperate developed and developing countries, smoke from household heating with solid fuels is another consequential but generally overlooked and underregulated problem (McGowan et al. 2002).

## Conclusions

The combustion of solid fuels for household cooking is an important contributor to ambient fine particulate air pollution (APM_2.5_) in many countries, accounting for > 10% of APM_2.5_ pollution in 7 regions housing > 50% of the global population in 2010. Regional proportions reach as high as 37% (sub-Saharan Africa); and the world as a whole, including many regions with no contribution from solid cooking fuel, averages about 12% of APM_2.5_ from household cooking with coal, wood, and other solid fuels. Within countries, it can be expected that the proportion of APM_2.5_ from household cooking is highest in rural areas where cooking with coal and biomass are most prevalent. The importance of this source of pollution extends to the regions with the two most populous countries (India in South Asia and China in East Asia), both with high ambient pollution levels; together these regions account for nearly 90% of the estimated global deaths from ambient air pollution that were attributed to household cooking with solid fuels. In terms of absolute concentrations, in two regions that face severe air pollution problems and are home to about 3 billion people, South Asia and East Asia, the estimated contribution of household cooking to APM_2.5_ pollution ranged from 7 to 9 μg/m^3^ in 2010.

Ambient air pollution remains a significant health, environmental, and economic problem around the world. China, India, and many other countries with emerging economies face daunting air pollution challenges. This problem is not confined to densely populated megacities, but is a feature of small cities and interurban areas as well (Brauer et al. 2012). Our results indicate one important reason: the persistence of solid fuel use for cooking. Such fuels emit substantial amounts of ambient air pollution, while also being a risk in the household environment. Globally, more households use solid fuels for cooking today than at any time in human history, even as the fraction of the total population using solid fuels continues to slowly fall (Bonjour et al. 2013).

More collaboration and coordination will be needed between the household energy and general air pollution communities, both at the research and policy levels to deal with this issue. Currently these communities act in essential isolation, as illustrated for example by the lack of ambient monitoring stations and reporting of pollution levels in rural areas in nearly all developing countries (Balakrishnan et al. 2011). In reality, both the household energy and air pollution communities have a stake in finding clean cooking fuels and clean cookstoves, which not only protect people in and around the households of the poor, who currently rely on polluting solid fuels, but also need to be part of national strategies to control ambient pollutions for the protection of all.

## Supplemental Material

(441 KB) PDF
