# Supplementary material for: Household Cooking with Solid Fuels Contributes to Ambient PM2.5 Air Pollution and the Burden of Disease
Source: Environ Health Perspect. 2014 Sep 5;122(12):1314–20. doi: 10.1289/ehp.1206340 (PMC4256045; doi:10.1289/ehp.1206340)
Supplement: (441 KB) PDF [file ehp.1206340.s001.508.pdf]

Environmental Health Perspectives doi:10.1289/ehp.1206340

Supplemental Material

Household Cooking with Solid Fuels Contributes to Ambient $PM_{2.5}$ Air Pollution and the Burden of Disease

Zoë A. Chafe, Michael Brauer, Zbigniew Klimont, Rita Van Dingenen, Sumi Mehta, Shilpa Rao, Keywan Riahi, Frank Dentener, and Kirk R. Smith

Model Methodologies

The GAINS data used in Equation 1 represent the $PPM_{2.5}$ attributable to cooking at the household level. They do not include, in either the numerator or denominator, secondary particle formation. The TM5-FASST data, used in Equation 2, include $PPM_{2.5}$ and associated secondary particles; they represent do not include dust or sea salt. To estimate the dust/salt increment, country- or regional-level estimates of combustion-derived $PM_{2.5}$ obtained from TM5-FASST were compared with country-level estimates of total $APM_{2.5}$ (including dust and sea salt) developed in Brauer et al.(Brauer et al. 2012) for the Global Burden of Disease project, using inputs from TM5-FASST as well as ground and satellite observations. Current air quality legislations are included for both 2005 and 2010 $PM_{2.5}$ estimates. While ambient air quality legislations do not necessarily have a large impact on cooking-related emissions, the total $APM_{2.5}$ in 2010 would have been higher if no legislation was assumed in the 2000-2010 period.

MESSAGE provides global, regional, and spatially explicit emissions of (CH4), sulfur dioxide (SO_2), nitrogen oxides (NO_X), carbon monoxide (CO), volatile organic compounds (VOCs), black carbon (BC), organic carbon (OC), and $PM_{2.5}$ (at a 1°x1° resolution). The downscaling methods for the socio-economic and demographic drivers as well as emissions are described in Grubler et al. (2007) and Riahi et al. (2011).

MESSAGE covers all greenhouse gas (GHG)-emitting sectors, including power plants, industry (combustion and process), road transport, households, international shipping and aviation, agricultural waste burning, and biomass burning (deforestation, savannah burning, and vegetation fires) for a full basket of greenhouse gases and other radiatively active gases.

To estimate the impacts of these spatially explicit emissions, atmospheric concentrations of average ambient population-exposure weighted anthropogenic $PM_{2.5}$ and also specifically the household-related fraction are further derived using the TM5 –FASST source-receptor model. Modeled $PM_{2.5}$ includes contributions from (i) primary $PM_{2.5}$ released from anthropogenic sources and forest fires, and (ii) secondary inorganic aerosols formed from anthropogenic emissions of SO_2, NO_x and NH_3 (including water vapor). The data are reported on a spatial level and are then aggregated by country and GBD region. (See Supplemental Material, Table 1.)

Population-weighted annual average ambient $PM_{2.5}$ concentration estimates ($APM_{2.5}$) were produced by overlaying $APM_{2.5}$ concentrations (from TM5-FASST, 1°x1° resolution) with high-resolution population maps (0.042°x0.042°). This allowed us to adjust the primary components of $PM_{2.5}$ concentration gradients within the 1°x1° gridcell using the underlying population gradients as a proxy. This method parametrizes the so-called urban increment and allows for a population-weighted $APM_{2.5}$ concentration which is higher than or equal to the 1°x1° area-averaged $PM_{2.5}$ concentration (Brauer et al. 2012; Rao et al. 2012).

References

Brauer M, Amann M, Burnett RT, Cohen A, Dentener F, Ezzati M, et al. 2012. Exposure assessment for estimation of the global burden of disease attributable to outdoor air pollution. Environ Sci Technol 46:652-660.

Grubler A, O'Neill B, Riahi K, Chirkov V, Goujon A, Kolp P, et al. 2007. Regional, national, and spatially explicit scenarios of demographic and economic change based on SRES. Technological Forecasting and Social Change 74:980-1029.

Rao S, Chirkov V, Dentener F, Dingenen R, Pachauri S, Purohit P, et al. 2012. Environmental modeling and methods for estimation of the global health impacts of air pollution. Environ Model Assess:1-10.

Riahi K, Rao S, Krey V, Cho C, Chirkov V, Fischer G, et al. 2011. RCP 8.5 - a scenario of comparatively high greenhouse gas emissions. Climatic Change 109:33-57.

Table S1. Regional Groupings Used in Global Burden of Disease 2010 (With Regional Population in 2010 of Countries Included in this Analysis and Total Population in Region). Note: countries that were not included in this analysis, because of data gaps, are shown in italics.

Region (population in millions)	Countries in region
Asia Central (81/81)	Armenia, Azerbaijan, Georgia, Kazakhstan, Kyrgyzstan, Mongolia, Tajikistan, Turkmenistan, Uzbekistan
Asia East (1,383/1,383)	China, Democratic People's Republic of Korea, *Hong Kong*
Asia Pacific High Income (181/181)	Brunei Darussalam, Japan, Republic of Korea, Singapore
Asia South (1,591/1,591)	Afghanistan, Bangladesh, Bhutan, India, Nepal, Pakistan
Asia Southeast (609/610)	Indonesia, Cambodia, Lao People's Democratic Republic, Sri Lanka, *Maldives*, Myanmar, Malaysia, Philippines, Thailand, *Timor-Leste*, Viet Nam
Australasia (26/26)	Australia, New Zealand
Caribbean (38/40)	Antigua and Barbuda, *Aruba*, Bahamas, Belize, Barbados, Cuba, Dominica, Dominican Republic, Grenada, *Guadeloupe,* Guyana, Haiti, Jamaica, *Martinique, Netherlands Antilles*, Saint Kitts and Nevis, Saint Lucia, Suriname, Trinidad and Tobago, Saint Vincent and the Grenadines
Europe Central (108/108)	Albania, Bulgaria, Bosnia and Herzegovina, Czech Republic, Croatia, Hungary, The former Yugoslav Republic of Macedonia, Montenegro, Poland, Romania, Serbia, Slovakia, Slovenia
Europe Eastern (206/206)	Belarus, Estonia, Lithuania, Latvia, Republic of Moldova, Russian Federation, Ukraine
Europe Western (414/414)	Andorra, Austria, Belgium, Switzerland, Cyprus, Germany, Denmark, Spain, Finland, France, United Kingdom, Greece, Ireland, Iceland, Israel, Italy, Luxembourg, *Monaco,* Malta, Netherlands, Norway, Portugal, San Marino, Sweden
Latin America Andean (53/53)	Bolivia, Ecuador, Peru
Latin America Central (230/230)	Colombia, Costa Rica, Guatemala, Honduras, Mexico, Nicaragua, Panama, El Salvador, Venezuela
Latin America Southern (61/61)	Argentina, Chile, Uruguay
Latin America Tropical (205/205)	Brazil, Paraguay
North Africa Middle East (473/478)	United Arab Emirates, Bahrain, Algeria, Egypt, Iran, Iraq, Jordan, Kuwait, Lebanon, Libyan Arab Jamahiriya, Morocco, Oman, Qatar, *Palestinian Territories*, Saudi Arabia, Syrian Arab Republic, Tunisia, Turkey, Yemen
North America High Income (348/348)	Canada, United States of America
Oceania (0/9)	*American Samoa, Cook Islands, Fiji, Micronesia, Guam, Kiribati, Marshall Islands, Micronesia, Niue, Nauru, New Caledonia, Palau, Papua New Guinea, Solomon Islands, Tonga, Tuvalu, Vanuatu, Samoa*
Sub-Saharan Africa Central (98/98)	Angola, Central African Republic, Democratic Republic of the Congo, Congo, Gabon, Equatorial Guinea
Sub-Saharan Africa East (357/357)	Burundi, Comoros, Djibouti, Eritrea, Ethiopia, Kenya, Madagascar, Mozambique, Mauritius, Malawi, Rwanda, Sudan, Somalia, Seychelles, United Republic of Tanzania, Uganda, Zambia
Sub-Saharan Africa Southern (70/70)	Botswana, Lesotho, Namibia, Swaziland, South Africa, Zimbabwe
Sub-Saharan Africa West (339/339)	Benin, Burkina Faso, Côte d'Ivoire, Cameroon, Cape Verde, Ghana, Guinea, Gambia, Guinea-Bissau, Liberia, Mali, Mauritania, Niger, Nigeria, Senegal, Sierra Leone, Sao Tome and Principe, Chad, Togo

Table S2. Regional Groupings Used in TM5-FASST.

Region	Countries in region
ARG	Argentina, Falkland Islands, Uruguay
AUT	Austria, Liechtenstein, Slovenia
BLX	Belgium, Luxembourg, Netherlands
CAN	Canada, Greenland
CHN	China, Hong Kong, Macau
EAF	Burundi, Central African Republic, Chad, Comoros, DR Congo, Djibouti, Eritrea, Ethiopia, Kenya, Madagascar, Mauritius, Reunion, Rwanda, Seychelles, Somalia, Sudan, Tanzania, Uganda
ESP	Gibraltar, Portugal, Spain
FRA	France, Andorra
GBR	Ireland, United Kingdom
GOLF	Bahrain, Iran, Iraq, Kuwait, Oman, Qatar, Saudi Arabia, United Arab Emirates, Yemen
GRC	Cyprus, Greece
IDN	Indonesia, Timor-Leste
ITA	Italy, Malta, Monaco, San Marino, Vatican City State
MEME	Israel, Jordan, Lebanon, Palestinian Territory, Syrian Arab Republic
MON	North Korea, Mongolia
MYS	Brunei Darussalam, Malaysia, Singapore
NDE	India, Maldives, Sri Lanka
NOA	Algeria, Libya, Morocco, Tunisia, Western Sahara
NOR	Iceland, Norway, Svalbard and Jan Mayen Islands
PAC	Fiji, French Polynesia, Guam, Kiribati, Marshall Islands, Micronesia, Nauru, New Caledonia, Niue, Norfolk Island, Northern Mariana Islands, Palau, Papua New Guinea, Pitcairn, Samoa, Solomon Islands, Tokelau, Tonga, Tuvalu, Vanuatu, Wallis and Futuna Islands
POL	Estonia, Latvia, Lithuania, Poland
RCAM	Anguilla, Antigua and Barbuda, Aruba, Bahamas, Barbados, Belize, Cayman Islands, Costa Rica, Cuba, Dominica, Dominican Republic, El Salvador, Grenada, Guadeloupe, Guatemala, Haiti, Honduras, Jamaica, Martinique, Montserrat, Netherlands Antilles, Nicaragua, Panama, Puerto Rico, Saint Kitts and Nevis, Saint Lucia, Saint Vincent and the Grenadines, Trinidad and Tobago, Turks and Caicos Islands, Virgin Islands (British), Virgin Islands (U.S.)
RCEU	Albania, Bosnia and Herzegovina, Croatia, Macedonia, Serbia and Montenegro
RCZ	Czech Republic, Slovakia
RIS	Kyrgyzstan, Tajikistan, Turkmenistan, Uzbekistan
RSA	Lesotho, South Africa, Swaziland
RSAM	Bolivia, Colombia, Ecuador, French Guiana, Guyana, Paraguay, Peru, Suriname, Venezuela
RSAS	Afghanistan, Bangladesh, Bhutan, Nepal, Pakistan
RSEA	Cambodia, Laos, Myanmar
RUS	Armenia, Azerbaijan, Georgia, Russian Federation
SAF	Angola, Botswana, Malawi, Mayotte, Mozambique, Namibia, Zambia, Zimbabwe
SWE	Denmark, Faroe Islands, Sweden
UKR	Belarus, Moldova, Ukraine
USA	Bermuda, Saint Pierre and Miquelon, United States
WAF	Benin, Burkina Faso, Cameroon, Cape Verde, Congo, Cote D'Ivoire, Equatorial Guinea, Gabon, Gambia, Ghana, Guinea, Guinea-Bissau, Liberia, Mali, Mauritania, Niger, Nigeria, Saint Helena, Sao Tome and Principe, Senegal, Sierra Leone, Togo

Table S3. Regional Groupings Used in GAINS.

Region	Countries in region
Former USSR (Asia)	Tajikistan, Turkmenistan, Uzbekistan
Middle East	Bahrain, Iran, Iraq, Jordan, Kuwait, Lebanon, Oman, Qatar, Saudi Arabia, Syrian Arab Republic, United Arab Emirates, Yemen
North Africa	Algeria, Libyan Arab Jamahiriya, Morocco, Sudan, Tunisia
Other Africa	Angola, Benin, Botswana, Burkina Faso, Burundi, Cameroon, Cape Verde, Central African Republic, Chad, Comoros, Democratic Republic of Congo, Congo, Cote d'Ivoire, Djibouti, Equatorial Guinea, Eritrea, Ethiopia, Gabon, Gambia, Ghana, Guinea, Guinea-Bissau, Kenya, Lesotho, Liberia, Madagascar, Malawi, Mali, Mauritania, Mauritius, Mozambique, Namibia, Niger, Nigeria, Rwanda, Sao Tome and Principe, Senegal, Seychelles, Sierra Leone, Somalia, Swaziland, Tanzania, Togo, Uganda, Western Sahara, Zambia, Zimbabwe
Other Latin America	Antigua and Barbuda, Bahamas, Barbados, Belize, Bolivia, Colombia, Costa Rica, Cuba, Dominica, Dominican Republic, Ecuador, El Salvador, French Guiana, Grenada, Guatemala, Guyana, Haiti, Honduras, Jamaica, Nicaragua, Panama, Paraguay, Peru, Puerto Rico, Saint Kitts and Nevis, Saint Lucia, Saint Vincent and the Grenadines, Suriname, Trinidad and Tobago, Uruguay, Venezuela

Table S4. Population and Emissions Data Used in the Analysis.

Region	Population 1990[a]	Population 2005[a]	Population 2010[a]	$PPM_{2.5\text{-}cook}$ 1990[b]	$PPM_{2.5\text{-}cook}$ 2005[b]	$PPM_{2.5\text{-}cook}$ 2010[b]	$PM_{2.5\text{-}hh}$ 1990[c]	$PM_{2.5\text{-}hh}$ 2005[c]	$PM_{2.5\text{-}hh}$ 2010[c]
High-income Asia Pacific	170	180	181	14%	5%	6%	12%	14%	12%
Central Asia	69	77	81	3%	0%	1%	23%	36%	21%
East Asia	1175	1344	1383	83%	85%	85%	34%	24%	18%
Southeast Asia	455	572	609	90%	89%	89%	28%	21%	18%
South Asia	1106	1473	1591	82%	83%	82%	21%	36%	32%
Australasia	20	24	26	4%	2%	2%	2%	5%	4%
Caribbean	31	37	38	83%	83%	83%	12%	11%	14%
Central Europe	112	109	108	0%	0%	0%	19%	31%	23%
Eastern Europe	223	212	206	0%	0%	0%	19%	32%	19%
Western Europe	381	407	414	0%	0%	0%	10%	16%	14%
Andean Latin America	39	50	53	83%	83%	83%	7%	10%	11%
Central Latin America	167	215	230	83%	83%	83%	8%	7%	9%
Southern Latin America	49	58	61	82%	81%	81%	13%	16%	18%
Tropical Latin America	154	193	205	83%	83%	83%	5%	8%	9%
High-income North America	284	332	348	0%	0%	0%	4%	10%	10%
North Africa and Middle East	318	432	473	34%	31%	31%	15%	15%	14%
Central sub-Saharan Africa	55	84	98	99%	99%	99%	6%	17%	19%
Eastern sub-Saharan Africa	211	314	357	97%	94%	94%	6%	16%	18%
Southern sub-Saharan Africa	52	68	70	95%	94%	93%	13%	34%	41%
Western sub-Saharan Africa	199	301	339	99%	99%	99%	10%	26%	30%
**World**	**5269**	**6483**	**6872**	**62%**	**65%**	**65%**	**20%**	**23%**	**21%**

[a]Population x 10^6. [b]Percent of primary $PM_{2.5}$ household emissions attributable to household cooking (GAINS). [c]Percent of combustion-derived emissions attributable to household cooking and heating (TM5-FASST).

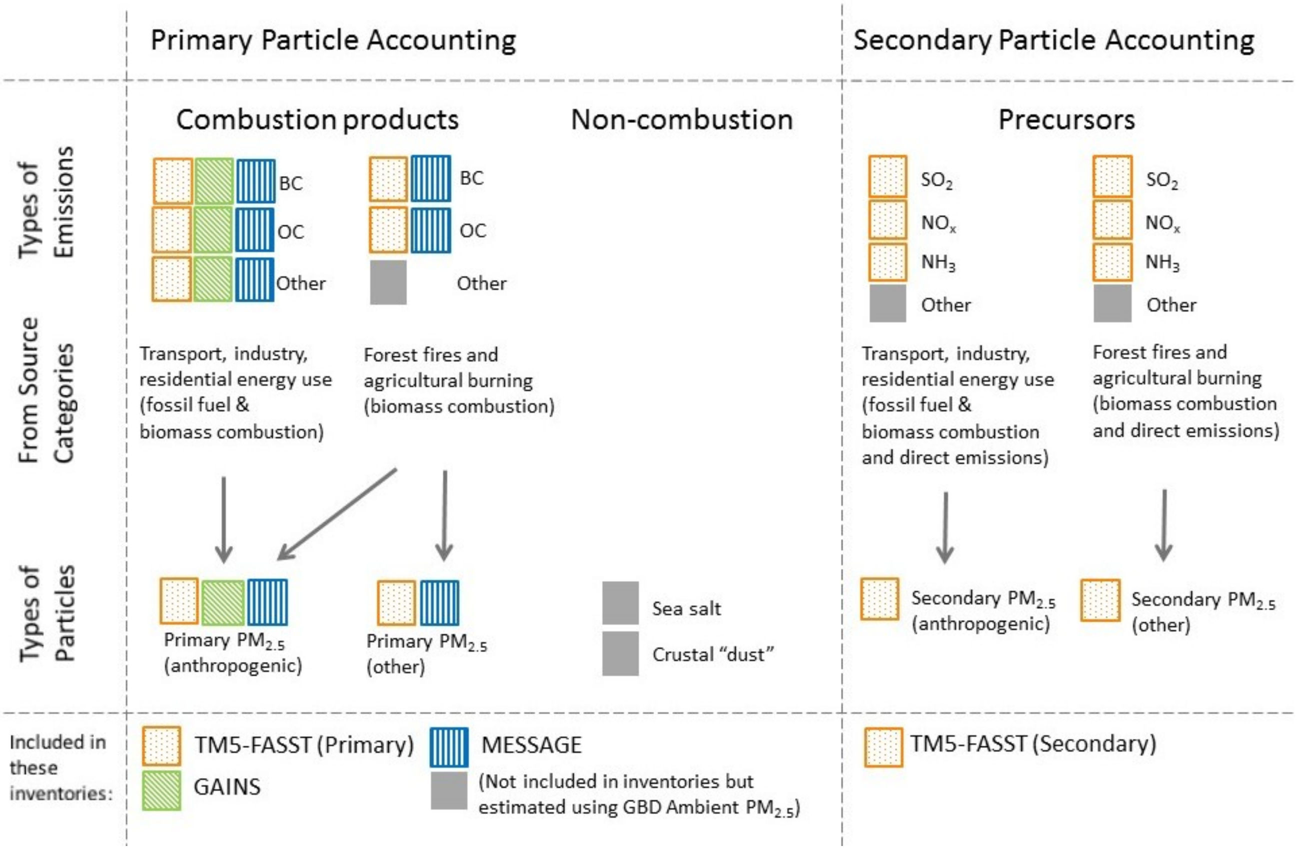

Figure S1. Emissions and particle coverage in the major databases and models used in this analysis. Note that sea salt, dust, and some secondary particle precursors are not included in the models used here; however, they are represented in the total ambient $PM_{2.5}$ concentrations calculate for GBD 2010, published in Brauer et al. (2012) and used in the final stages of the analysis presented in this analysis.
